# Crossing effect for improving egg production traits in chickens involving local and commercial strains

**DOI:** 10.14202/vetworld.2020.407-412

**Published:** 2020-03-03

**Authors:** Mostafa Ahmed Soliman, Mohamed Hassan Khalil, Karim El-Sabrout, Mostafa Kamel Shebl

**Affiliations:** Department of Poultry Production, Faculty of Agriculture (El-Shatby), Alexandria University, Alexandria, Egypt

**Keywords:** additive effect, crossbreeding, heterosis, laying hens, reciprocal effect

## Abstract

**Aim:**

This study aimed to contribute to the productivity improvement of the local chickens by enhancing their egg production traits using a crossbreeding program between Alexandria (local strain) and Lohmann White (commercial strain).

**Materials and Methods:**

One thousand two-hundred and eighty-five 4-week-old chicks from two strains: Alexandria local strain (AA) and Lohmann White commercial strain (LL) and their reciprocal crosses obtained from 16 males and 160 females, were used to produce four genetic groups (AA, LL, AL, and LA). Differences among genotypes, direct additive, heterosis, and reciprocal effects were investigated regarding the following traits: Body weight at 4 and 8 weeks and at the age of sexual maturity, age at sexual maturity, egg production, average egg weight, and egg mass during the first 90 days of laying.

**Results:**

Statistically significant effects of the genotypes were observed on traits studied. Analysis of direct additive effects showed that AA was superior as a sire strain for improving body weight at early age. For egg traits (age at sexual maturity, egg production, average egg weight, and egg mass), LL was better as a sire strain to improve these traits. Significant positive heterosis percentages were observed for body weight. The crosses (AL and reciprocal) were significantly superior in egg traits (egg production, average egg weight, and egg mass) compared to the local strain. The cross (LA) laid significantly earlier than the local strain. Analysis of reciprocal effects cleared that the local strain could be used as a strain of dam to improve body weight and egg traits.

**Conclusion:**

Crossing improved egg production, egg weight, and egg mass in hybrids compared to the local strain.

## Introduction

Local chickens are known to own desirable characteristics such as resistance to some diseases, wonderful meat flavor, and taste [1,2]. Therefore, the consumption of meat products from local chickens has increased in countries of Africa and developing countries. Local chicken strains have better survival than the commercial hybrid strain under local production conditions, but they had poor egg production [3]. Fayoumi is an Egyptian breed of chicken and is well suited to hot climates and free-range management [4]. Fayoumi breed could be incorporated in the crossbreeding programs to improve the genetic resistance to Newcastle disease [5] and a better strategy to upgrade the poor performance of indigenous chicken populations [6]. Commercial hens breeding systems widely use crossbreeding programs to exploit heterosis [7]. Heterosis (hybrid vigor) is the extent to which the performance of a crossbred in one or more traits is better than the average performance of the two parents. The effectiveness of crossing for genetic enhancement of quantitative characteristics such as egg production in the chicken was proven in the literature.

Genetic crossing is one of the important breeding programs that play a major role in the improvement of the chicken’s performance. Improving the productivity of local strains is considered one of the priorities of the Egyptian poultry industry [8]. The synthesis of new hybrid line is essential for achieving high productivity from laying hens.

The current study aimed to contribute to the productivity improvement of the local chickens by enhancing their egg production traits using crossbreeding program between Alexandria (local strain) and Lohmann White (commercial strain).

## Materials and Methods

### Ethical approval

The approval from the Institutional Animal Ethics Committee to carry out this study was not required as no invasive technique was used.

### Study design

The experimental work of the present study was conducted at the Poultry Research Center, Poultry Production Department, Faculty of Agriculture, Alexandria University, Egypt, during the breeding season 2017/2018. Two strains of chickens were used in the present study as follows:

1. Local Egyptian strain: Alexandria chickens (AA) were obtained in 1958 by crossing between four strains of chickens (White Leghorn, Rhode Island Red, Plymouth Rock, and Fayoumi) [9]
2. Commercial Lohmann White (LL) was obtained from the Valley Company.

### The mating plan

A total of 160 hens from the Alexandria (AA) and Lohmann (LL) strains (80 each) were distributed in 16 individual breeding pens with sex ratio of 1:10. At 50 weeks of age, hens of each strain were divided at random into two equal groups. The first group was mated with roosters from the same strain, whereas the second, with roosters of the alternative strain to obtain reciprocal crosses. The breeding plan permitted the simultaneous production of pure strains: Alexandria × Alexandria (AA), Lohmann × Lohmann (LL), and crosses: Alexandria × Lohmann (AL) and Lohmann × Alexandria (LA). In listing the crosses, the male parent is presented first. Mating within strains was at random except that sib-mating was avoided. The pure strains and reciprocal crosses offspring were obtained in 6 weekly hatches.

### Flock husbandry

The identified eggs were pedigreed for each dam through trap nesting. All experimental parents and hatching eggs received the same managerial treatments for all strains. Feed and water were provided *ad libitum*. At hatching, the chicks were pedigreed, wing banded, and brooded in floor brooders, then transferred to the rearing houses with equal conditions on deep litter. At 20 weeks of age, the pullets were assigned to individual laying cages. Chicks were feed during rearing, growing, and laying periods on diet containing 21%, 18%, and 16% crude protein, respectively. The pullets were exposed to light for 16 h/day from 20 weeks of age till the end of the experimental period. All birds were treated and medicated similarly throughout the experimental period under the same managerial and climatic conditions. For chicks, the following traits were individually recorded for both sexes: Body weight (g) at 4 and 8 weeks of age for both sexes. For pullets, the following traits were individually recorded: Age of sexual maturity (d), body weight at sexual maturity (g), egg number (egg), average egg weight (g), and egg mass (g) during the first 90 days of laying.

### Statistical analysis

Data were analyzed for variation between the genotypes using the general linear model of IBM SPSS statistic 24 software [10]. Differences were tested for significance using Duncan test [11]. For body weight, the following linear model was tested to analyze the data:

Y_ijkl_ = U + G_i_ + H_j_ + S_k_ + GH_ij_ + GS_ik_ + HS_jk_ + GHS_ijk_+ e_ijkl_

Where: Y_ijkl_ = the observation on the genotype, U = the overall mean, G_i_ = the fixed effect of i^th^ genotype, H_j_ = the fixed effect of j^th^ hatch, S_k_ = the fixed effect of k^th^ sex, GH_ij_, GS_ik_, HS_jk_, GHS_ijk_ = the interaction between the fixed effects, and e_ijkl_ = random error.

For egg traits, data were analyzed using the previous general linear model without considering sex effect, which was excluded from the model.

### Estimation of crossbreeding components

Estimates of direct additive effect, direct heterosis, and reciprocal effect for all traits were calculated according to Dickerson [12,13] using the software package of IBM SPSS statistic 24 [10].

1. Pure strain difference: [(AA × AA – LL × LL)]
2. Direct additive effect: [(AA × AA + AA × LL) – (LL × LL + LL × AA)]/2
3. Direct heterosis: [(AA × LL + LL × AA) – (AA × AA + LL × LL)]/2
4. Reciprocal effect: [(LL × AA) – (AA × LL)]/2

The heterosis (H%) was calculated according to the formula [14]:

H%= [F_1_ − (P_1_ + P_2_)/2]/[(P_1_ + P_2_)/2] × 100

Where:

 F_1_: Average values of traits of hybrid strains

 Р_1_, P_2_: Average values of traits of original strains.

## Results

Comparisons between genotypes showed statistically significant differences for all traits studied (Tables-1 and 2).

**Table-1:** Means and SE of body weight by age, genetic group, and sex.

Body weight (g) at	Genetic group								Sig.	Sex				Sig.
	
	AA		LL		AL		LA			Male		Female		
					
	n	Mean±SE	n	Mean±SE	n	Mean±SE	n	Mean±SE		n	Mean±SE	n	Mean±SE	
4 weeks	274	237.50^a^±2.71	321	172.12^c^±2.13	425	223.81^b^±2.09	265	240.91^a^±2.43	****	521	222.61±1.77	764	211.83±1.44	****
8 weeks	180	649.54^a^±11.77	79	510.06^b^±9.76	244	635.72^a^±7.76	155	621.50^a^±12.14	****	244	679.80±9.12	414	573.32±8.43	****
Sexual maturity	61	1669.16^b^±32.64	20	1829.25^a^±4.73	55	1649.72^b^±28.21	43	1563.74^b^±35.42	****					

**** p≤0.0001.

a,b,c Different letters within a row for genetic group indicate statistically significant differences. AA=Alexandria × Alexandria, LL=Lohmann × Lohmann, AL=Alexandria male × Lohmann female, LA=Lohmann male × Alexandria female, SE=Standard error

**Table-2:** Means and SE of egg traits by genetic group.

Traits	Genetic group								Sig.

	AA		LL		AL		LA		
			
	n	Mean±SE	n	Mean±SE	n	Mean±SE	n	Mean±SE	
Age at sexual maturity (d)	61	182.78^a^±2.50	20	151.40^c^±0.86	55	180.01^ab^±2.64	43	172.83^b^±2.31	****
Egg number (egg)	54	33.94^c^±1.91	20	59.00^a^±0.78	51	46.37^b^±2.20	38	42.28^b^±2.30	****
Average egg weight (g)	54	47.47^c^±0.63	20	57.55^a^±0.40	51	53.14^b^±0.64	38	52.29^b^±0.88	****
Egg mass (g)	54	1630.67^c^±100.26	20	3394.82^a^±47.35	51	2452.69^b^±117.47	38	2231.51^b^±137.35	****

**** p≤0.0001. AA=Alexandria × Alexandria, LL=Lohmann × Lohmann, AL=Alexandria male × Lohmann female, LA=Lohmann male × Alexandria female,

a,b,c Different letters within a row for genetic group indicate statistically significant differences

The local strain, Alexandria (AA), resulted significantly in higher body weight at 8 weeks of age compared to the commercial Lohmann strain (LL) and the other crossbreds (AL and LA). On the other hand, the commercial strain (LL) significantly surpassed the local strain (AA) and both crossbreds (AL and LA) in body weight at sexual maturity (1829.25 vs. 1669.16, 1649.72, and 1563.74 g), respectively.

Sex had a significant effect on body weight at 4 and 8 weeks of age. Males were significantly heavier than females at all ages.

The local strain (AA) laid first egg significantly later compared to the commercial strain (LL), (182.78 vs. 151.40 d). However, the crossbred (LA) laid significantly earlier compared to the local strain (AA) (172.83 vs. 182.78 d).

Both crossbreds (AL and LA) significantly laid more eggs than the local strain (AA) (46.37 and 42.28 vs. 33.94 eggs), but they were consistently less than the commercial strain (LL) (59.00 eggs).

Both crossbreds (AL and LA) significantly laid heavier eggs than the local strain (AA) (53.14 and 52.29 vs. 47.47 g), but they were consistently less than the commercial strain (LL) (57.55 g). The same trend was observed for egg mass. The crossbred (AL and LA) significantly laid egg mass (2452.69 and 2231.51 g) more than the local strain (1630.67 g), but they were less than the commercial strain (3394.82 g).

### Pure strain difference

Comparisons between the purebred genotypes showed statistically significant differences for all traits studied (Table-3). The linear contrasts evidenced that the local strain (AA) had significantly superior performance in terms of body weight at 4 and 8 weeks of age by 65.37 and 139.48 g, respectively, compared with the commercial strain (LL). While, regarding body weight at sexual maturity, the commercial strain was significantly heavier by 160.08 g more than the local strain. In addition, the commercial strain laid first egg significantly earlier by 31.38 d, higher egg production by 25.05 eggs, and heavier eggs and egg mass by 10.08 and 1764.14 g, respectively, in the first 90 days of laying compared to the local strain.

**Table-3:** Crossing estimates (±standard error) of body weight and egg traits.

Traits	Pure strains difference	Direct additive effect	Heterosis		Reciprocal effect

			Contrast	%	
Body weight (g) at					
4 weeks	65.37****±3.42	24.14****±2.36	27.55****±2.36	13.45****	8.54****±1.62
8 weeks	139.48****±18.39	76.85****±11.55	48.81****±11.55	8.41****	−7.11^ns^±6.99
Sexual maturity	−160.08**±56.91	−37.05^ns^±36.22	−142.48****±36.26	−8.15****	−42.99^ns^±22.48
Age at sexual maturity (d)	31.38****±4.51	19.28****±2.87	9.33***±2.87	5.58***	−3.59*±1.78
Egg number (egg)	−25.05****±3.63	−10.48****±2.34	−2.14^ns^±2.34	−4.62^ns^	−2.04^ns^±1.48
Average egg weight (g)	−10.08****±1.20	−4.61****±0.77	0.20^ns^±0.77	0.39^ns^	−0.42^ns^±0.49
Egg mass (g)	−1764.14****±198.39	−771.48****±128.20	−170.65^ns^±128.20	−6.79^ns^	−110.58^ns^±81.21

* p≤0.05,

** p≤0.01,

*** p≤0.001,

**** p≤0.0001, ns=Not significant

### Direct additive effect

Direct additive effects were statistically significant for all traits studied except body weight at sexual maturity (Table-3). Body weight at early ages (4 and 8 weeks) of chicks sired by the purebred (AA) was significantly superior more than those sired by the purebred (LL) by 24.14 and 76.85 g, respectively. The opposite trend was observed in later age for body weight at sexual maturity although it was statistically not significant. Compared to AA sired chickens, LL sired chickens laid eggs significantly earlier by 19.28 d and were significantly superior in egg number by 10.48 egg, egg weight by 4.61 g, and egg mass by 771.48 g in the first 90 days of laying.

### Heterotic effects

Heterotic effects for the studied traits are shown in Table-3. Significant positive heterosis contrasts (i.e., the value of the crosses was higher than the average of the parental strains) were observed in body weight at 4 and 8 weeks of age with percentages of 13.45 and 8.41%, respectively. In contrast, significant negative heterosis (−8.15%) for body weight at sexual maturity was observed (i.e., the crosses were less in body weight at sexual maturity than the average of the parental strains). Significant positive heterosis (5.58%) for age at sexual maturity was observed. Moreover, negative and non-significant heterosis percentages were observed for egg number (−4.62%) and egg mass (−6.79%). In contrast, positive and non-significant heterosis percentage was observed for average egg weight (0.39%).

### Reciprocal effect

Reciprocal effects are shown in Table-3. Body weight of chicks at early age (4 weeks) mothered by AA strain was significantly superior to those mothered by LL strain. The changes of this trait with age change the level of significance. No difference between reciprocal crosses of AA and LL strains was observed for body weight at 8 weeks of age and at sexual maturity. Age at sexual maturity of chickens mothered by the local strain (AA) was significantly superior to those chickens mothered by the commercial strain (LL). On the other hand, reciprocal effects for egg number, egg weight, and egg mass were not significant, i.e., both local (AA) and commercial (LL) chicken strains could be used as strain of dam.

## Discussion

Significant differences between the local and commercial strains of chickens for body weight and egg traits were supported in the literature [15-19].

The significant effect of direct additive for body weight indicates that AA sired chicks were significantly superior in BW, which leads to conclude that the local strain (AA) could be used as a sire strain to improve this trait. Additive genetic variation as indicated by significant differences between breeds was large for body weight. Such variation was expected as it has been shown large gains in body weight. Similar studies reported that additive genes had a positive effect on growth body [20,21]. Iraqi [22] showed that direct additive effect for growth traits was significant for all body weights. Khalil *et al*. [23] found that direct additive effect ranged from 4.9% to 10.2% for body weights, while Amin *et al*. [24] reported that additive effects of the growth traits were not significant.

The significant effect of direct additive for egg traits (age at sexual maturity, egg number, egg weight, and egg mass) indicates that LL sired chickens resulted in favorable effects, which leads to conclude that the commercial strain (LL) could be used as a sire strain to improve these traits. Additive genetic variation in age at sexual maturity was reported in the literature. Khalil *et al*. [15] reported that percentages of direct additive effect were negative (−1.9%) for ASM, in the cross of White Leghorn × Baldi Saudi.

Additive genetic variations as indicated by significant differences between strains were large for egg production, egg weight, and egg mass. Such variation was expected and can be made by selection. Iraqi [22] reported that direct additive effect was 5.54 eggs for EN90D in a crossbreeding experiment between two Egyptian strains of chickens, namely, Mandarah and Matrouh. Nawar and Abdou [25] showed that the percentage of direct additive effect was –12.5% for EN90D when crossed R.I.R sires to Fayoumi dams. Khalil *et al*. [15] found that the percentage of direct additive effect was 26.5% for EN in the cross of White Leghorn × Baldi Saudi. Significant positive heterosis for body weight at early ages (4 and 8 weeks) supported the superiority of hybrids over the original breeder strains. In addition, crossing between the local strain (AA) and the commercial strain (LL) in this study improved age at sexual maturity, egg number, average egg weight, and egg mass in the hybrids (AL and LA) compared to the local strain. Both crosses (AL and LA) significantly laid more eggs than the local strain (AA) (46.37 and 42.28 vs. 33.94 eggs), but they were consistently less than the commercial strain (LL) (59.00 eggs). This may be related to the age of sexual maturity, which occurred earlier in the commercial hens (LL) than in local strain (AA). Abou El-Ghar *et al*. [3] concluded that this could be attributed to increased number of produced eggs together with the lower age of sexual maturity. In addition, the cross (AL) between AA males and LL females was statistically equal in terms of egg production to those of reciprocal cross (LA).

Heterosis or non-additive genetic variation has been observed in some chicken crosses, in which the parents differed greatly in ASM and egg production [26]. They reported significant differences between these strains for non-additive genetic effects to warrant the use of crossbreeding for the improvement of ASM and egg production. This could be attributed to the fact that crossbred often exhibit heterosis which often shows the existence of non-additive effects [27].

After breeding local hens with Lohmann Brown and Leghorn, some researchers reported that heterosis for the age of sexual maturity varied from –25% to 11.5% [26]. Fairfull *et al*. [28] reported that both dominance and epistasis played a significant role in heterosis for egg production traits. The heterosis for egg production reported in literature is highly variable, as it depends on the nature and degree of differences among strains, but it is often around 10% or greater [14].

The low heterotic values for egg weight trait in this study could suggest that egg weight of the base flock used was mostly governed by additive and residual gene effects. This is consistent with the report of Fairfull [14] and Groen *et al*. [29] that heterosis for egg weight was low and ranged from 0% to 5%. The low heterosis for this trait further agrees with the findings of Udeh and Omeje [30] and Yahaya *et al*. [31].

Reciprocal effect was significant for body weight at early age (4 weeks) and age at sexual maturity. However, no difference between reciprocal crosses of AA and LL strains for the other traits studied. Significant reciprocal effects for body weight were reported by Emad [32] in the diallel crossing of Saso, Italian, and Mandarah chickens at different ages. Age at sexual maturity of chickens mothered by the local strain (AA) was significantly superior to those chickens mothered by the commercial strain (LL). Therefore, it may be effective to use AA as a strain of dams in crossbreeding programs for producing chickens with earlier age in laying eggs. In other words, chickens produced through the crossing LL males with the AA females had an advantage over their reciprocal cross for ASM. Maternal effect inheritance may be the more plausible explanation for the reciprocal effect observed for ASM. An evidence for significant reciprocal effect for age at sexual maturity was obtained by Munisi *et al*. [18].

The lack of difference observed between the crosses (cross and reciprocal cross) for egg number, egg weight, egg mass, and body weight at sexual maturity in this study could be attributed to the absence of sex-linked and/or maternal effect in intercrossing of the strains involved. A non-significant difference between the cross and reciprocal cross had been reported in some studies [31,33].

## Conclusion

The results demonstrated a statistically significant effect of the genotype on all traits studied. Based on the analysis of direct additive effects, it could be concluded that the local strain (AA) was superior as a sire strain for improving body weight. However, for egg traits (age at sexual maturity, egg number, average egg weight, and egg mass), the commercial strain (LL) was better as a sire strain to improve these traits. Significant positive heterosis percentages were observed for body weight at earlier ages (4 and 8 weeks) which supported the superiority of hybrids over the original breeder strains. Although heterosis percentages were not significant for egg traits (egg number, average egg weight, and egg mass), both crosses (AL and LA) were significantly superior regarding these traits compared to the local strain (AA). In addition, the cross (LA) laid significantly earlier than the local strain (AA) and later than the commercial strain (LL). Regarding heterosis, crossing improved egg production and egg weight and mass in hybrids compared to the local strain. Regarding reciprocal effects, the local strain (AA) could be used as strain of dam to improve growth and egg traits.

## Authors’ Contributions

MAS: Helped in the practical experiment, drafted the manuscript and analyzed the data. MHK: Designed the study, supervision, helped in the practical experiment, and drafted the manuscript. KE: Supervised the study, drafted the manuscript, and helped in the practical experiment. MKS: Designed and supervised the study, drafted the manuscript and analyzed the data. All authors read and approved the final manuscript.
